# Anorexia and Starvation Related Duodenal Villous Atrophy in an Adult Patient

**DOI:** 10.7759/cureus.18536

**Published:** 2021-10-06

**Authors:** Ethan Tamlyn, Debashish Das

**Affiliations:** 1 Gastroenterology, Leicester Medical School, Leicester, GBR; 2 Gastroenterology, Kettering General Hospital, NHS Foundation Trust, Kettering, GBR

**Keywords:** villous atrophy, non-celiac, metabolic, malnutrition, non-coeliac

## Abstract

Coeliac disease is the most well-known cause of villous atrophy in duodenal biopsies. Other well recognised causes of villous atrophy include infection with *Giardia* *duodenalis* and HIV, peptic duodenitis, drug-induced enteropathy, common variable immunodeficiency, Crohn’s disease, Whipple’s disease, small intestinal bacterial overgrowth, eosinophilic gastroenteritis, tropical or collagenous sprue and autoimmune enteropathy. While mucosal adaptation due to malnutrition, leading to villous atrophy, has been reported in paediatric populations in Africa and South America, in hibernating animals and animal models of experimental starvation, there is very little literature on adult human subjects. We report a case of a 76-year-old gentleman, presenting with chronic metabolic acidosis and anorexia who was found to have villous atrophy on duodenal biopsy, in the absence of any evidence of coeliac disease or other non-coeliac enteropathy. The nutritional state improved with correction of the underlying metabolic abnormality, and serial endoscopic assessment showed improvement in the villous atrophy. We discuss the relationship between malnutrition and villous atrophy, and suggest a potential workup for non-coeliac villous atrophy.

## Introduction

In patients presenting with unformed stools or weight loss, a finding of villous blunting and mucosal atrophy on duodenal biopsy would point towards a diagnosis of coeliac disease in the vast majority of cases. Other possible causes of villous atrophy, albeit much less common, include a wide spectrum of conditions such as infection with *Giardia duodenalis* and HIV, peptic duodenitis, drug-induced enteropathy, common variable immunodeficiency (CVID), Crohn’s disease, Whipple’s disease, small intestinal bacterial overgrowth (SIBO), eosinophilic gastroenteritis, tropical or collagenous sprue and autoimmune enteropathy. It can also simply be an artifactual finding, depending on the plane in which the biopsy has been taken and processed [1,2].

Here we present the case of an adult patient, who was found to have villous atrophy on duodenal biopsy, but for whom coeliac and the other non-coeliac enteropathies listed above were conclusively excluded. The relationship between this patient’s malnutrition and his intestinal atrophy, which was shown to improve following the correction of his metabolic acidosis leading to restitution of his oral nutritional intake, makes for an interesting discussion of how villous atrophy can be worked up and managed. The longitudinal follow-up with serial biopsies over a period of several months, alongside incremental oral intake and the improvement in nutritional status, is unique, and represents a rarely reported aetiology for this common biopsy finding.

## Case presentation

Mr H, a 76-year-old retired school teacher, was referred by his general practitioner to the gastroenterology service in mid-2016 with iron deficiency anaemia and weight loss. His significant past medical history included urinary bladder cancer, which was definitively treated by radical cystoprostatectomy and ileal conduit surgery in mid-2015. He was referred to the cardiology services at the end of 2015 due to shortness of breath and had normal results on chest x-ray, electrocardiography and echocardiography and no explanation was found to account for his “breathlessness”. At the presentation to the gastroenterology clinic, he described feeling nauseous, lack of appetite, and a loss of more than 10% of his body weight over the preceding 6 months. He reported feeling dyspneic and lethargic, and passing unformed stools since his operation, but denied any bleeding. Blood tests showed iron deficiency anaemia, low folate level with macrocytosis, low phosphate and borderline low calcium levels and raised parathyroid hormone level. Testing for coeliac disease using anti-tissue transglutaminase IgA antibodies was negative with a normal IgA level. A review of historical test results showed peri-operative anaemia, partly corrected after iron supplementation, and a negative Bowel Cancer Screening Programme stool test result from 2 years ago. Stool cultures, tests for ova, parasites and cysts, and *Clostridium difficile* toxin were all negative. CT-colonography showed a normal appearance except for some diverticulosis involving the sigmoid colon. Apart from the expected post-operative changes and an incisional hernia affecting the anterior abdominal wall, there was no suggestion of an extracolonic mass lesion. Upper gastrointestinal endoscopy was visibly normal but biopsies were taken from the proximal duodenum.

While awaiting the reporting of the duodenal biopsy and outpatient clinic follow-up, Mr H progressively became cachectic with increasing nausea and anorexia and started showing some cognitive changes. He was admitted to the hospital, as he could hardly tolerate any oral intake. There was tachypnoea, disorientation and apathy, and he was struggling to make any coherent conversation. On admission, he was found to be in profound metabolic acidosis with hyper-chloremia, and significantly reduced renal function. The additional blood results are shown in Table 1.

**Table 1:** Patient’s blood results on admission to hospital in 2016. eGFR - Estimated glomerular filtration rate, MDRD -  Modification of Diet in Renal Disease, CRP - C-reactive protein

Parameter	Patient’s result	Normal range
Hemoglobin	82 g/L	(130–180)
Serum albumin	32 g/L	(35-50)
Serum globulin	19 g/L	(20-30)
Serum ferritin	292 µg/L	(15–300)
Serum vitamin B12	219 ng/L	(160–760)
Serum folate	1.3 µg/L	(2.0–11.0)
Serum sodium	136 mmol/L	(137–144)
Serum potassium	4.1 mmol/L	(3.5–4.9)
Serum chloride	113 mmol/L	(95–107)
Serum bicarbonate	9 mmol/L	(20–28)
Serum urea	26.4 mmol/L	(2.5–7.0)
Serum creatinine	202 µmol/L	(60–110)
eGFR (MDRD)	29.8 mL/min/1.73 m2	(>60)
Serum corrected calcium	1.87 mmol/L	(2.20–2.60)
Serum phosphate	0.64 mmol/L	(0.80–1.45)
Serum CRP	<5 mg/L	(<10)
Plasma lactate	1.5 mmol/L	(0.6–1.8)
Serum thyroid-stimulating hormone	1.88 mU/L	(0.4–5.0)

Ultrasound scan of the abdomen showed both kidneys to be of normal appearance, with no hydronephrosis or hydroureter, and a CT scan of the head revealed no overt abnormalities. A repeat CT scan of the chest, abdomen and pelvis, with contrast, showed normal lung fields, no filling defects in the pulmonary arteries, normal configuration of the ileal conduit with the parastomal hernia and a midline incisional hernia containing small and large bowel loops but no features of incarceration or strangulation. No enhancing mass lesion was noted.

The patient was started on oral sodium bicarbonate replacement 500mg three times a day, to correct his metabolic acidosis. Within a few days the clinical picture rapidly changed; cognitive changes and anorexia improved, tachypnoea settled, the patient started tolerating oral intake and began to mobilise independently. Correction of electrolytes like phosphate, magnesium and potassium was done as needed with regular monitoring for re-feeding changes. The hyperchloremic metabolic acidosis was corrected with the continued use of sodium bicarbonate tablets. He was discharged, having significantly improved, after a hospital stay of three weeks.

While he was an inpatient, the duodenal biopsy was reported as showing villous atrophy with moderate blunting of the villi. The lamina propria showed a moderately dense, mixed acute and chronic inflammatory cell infiltrate with some crypt hyperplasia. The overlying surface epithelium also showed infiltration by mixed inflammatory cells including scattered lymphocytes and neutrophil polymorphs. No dysplasia or malignancy was found. However, given the details of the case further serological and clinical correlation was advised. Repeat tests for anti-tissue transglutaminase IgA antibodies and anti-endomysial IgA antibodies were negative. HLA- DQ2 and DR8 genotypes were negative, thus coeliac disease was conclusively ruled out. Given the cognitive changes noted at admission, the possibility of Whipple’s disease was considered and Periodic acid-Schiff and Periodic acid-Schiff-diastase stains were carried out. However, no convincing Periodic acid-Schiff (PAS)-positive macrophages were identified and *Tropheryma whipplei* DNA PCR was negative on both blood and biopsy samples. As the cognitive changes resolved rapidly after correction of the metabolic acidosis, the cerebrospinal fluid study was not pursued. HIV testing was negative. The serum immunoglobulin electrophoresis showed normal levels of immunoglobulin IgG, IgM and IgA. A review of his medication history revealed none of the drugs associated with drug-induced villous atrophy, were involved at any point. While he had had previous small bowel surgery, there were no real blind loops and his serum folate was low so SIBO was not considered as the cause here. Whilst the antinuclear antibody test was positive, extractable nuclear antigen (ENA) antibodies and anti-double-stranded DNA antibodies were both negative. Anti-enterocyte IgG and anti-goblet-cell IgG antibodies were also negative.

Following discharge from hospital and with continued clinical improvement from correction of his acidosis with sodium bicarbonate tablets, his nausea and anorexia resolved, his oral intake increased and he started to gain weight. At follow-up clinic review, given that we did not feel we had a full explanation of the villous atrophy, we arranged for repeat duodenal biopsies. The histology showed an improvement in the villous blunting, with some crypt elongation remaining but no increase in intraepithelial lymphocytes.

When the patient was seen in the clinic later that year, he reported feeling the best he had felt since his original cystoprostatectomy, and the anaemia and weight loss had fully corrected. A further upper gastrointestinal endoscopy was arranged, two years since the first one, and duodenal biopsies showed nearly normal duodenal mucosa, with villous architecture within normal limits and certainly improved in comparison with his previous biopsies.

The basic therapeutic intervention that had been instituted in this patient was the sodium bicarbonate tablets: by correcting the metabolic acidosis, his clinical condition dramatically improved and his appetite increased. We postulate that this patient’s villous atrophy was driven by a significant reduction in oral food intake and by reversing the starvation, the villous atrophy reverted as shown on longitudinal follow-up with serial duodenal biopsies. While this phenomenon has been demonstrated in animal experiments and in malnutrition in paediatric populations, we did not come across reported cases in adult populations where the recovery of the villous atrophy has been demonstrated with longitudinal follow-up.

## Discussion

This case provides an example of the phenomenon of intestinal mucosal adaptation due to malnutrition, a less common but very likely under-reported cause of villous atrophy. It has been well established that prolonged periods of poor enteral intake can lead to intestinal failure via mucosal atrophy [3]. Animal studies, in particular of hibernatory species, have also shown that intestinal mass and villous architecture are both reduced during periods of prolonged fasting [4]. Similar changes have also been shown in experimental starvation in animals [5]. Studies conducted among pediatric populations, particularly in the African and South American continents, have reported impaired absorption and intestinal mucosal maladaptation associated with malnutrition [6]. Studies of protein-calorie malnourished children have shown significant malabsorption that improves over time with correction of the nutritional deficit [7]. Similarly, the duodenojejunal mucosa of such children has been shown to have reduced thickness and abnormal villous architecture which improves with nutritional recovery [8]. The similarity of changes in duodenal mucosa to that found in coeliac disease has also been identified, particularly in children and infants with kwashiorkor malnutrition [9,10].

The adaptive re-sizing of the intestine is one of the well recognised physiological responses to tissue demands. Villous atrophy has also been reported in patients receiving total parenteral nutrition (TPN), even when their overall nutritional intake was adequate, but their enteral intake was significantly reduced or completely absent [3]. However, it is worth noting that there is some debate on whether or not the institution of TPN is the cause of this atrophy, and more recent evidence from studies in critical care settings, suggests that all-in-one parenteral nutrition can be safe if used carefully [11,12].

The changes of the atrophy of villi and increased intestinal permeability have been shown to start rapidly, as soon as four days after stopping enteral feeding, indicating a rapid response of intestinal stem cells to nutrient availability [13]. In experimental studies in rats, it has also been shown that diverting the flow of food from a segment of small bowel results in mucosal atrophy in the isolated segment, indicated by altered histology, changes in differentiation marker expression, and extracellular signal-regulated kinase (ERK) signaling, even in animals that are otherwise able to maintain adequate enteral nutrition [14]. This suggests that direct contact with luminal chyme is required to sustain the mucosa. The mucosal health and integrity are possibly maintained due to the direct trophic effect of luminal nutrients themselves. In addition, it is postulated that the physical forces that the mucosa is exposed to, either directly by peristaltic compression against the non-compressible liquid contents of the bowel or indirectly by villus motility, may also contribute to the health of the mucosa [3].

In this gentleman’s case, the cause of hyperchloremic metabolic acidosis was thought to be his ileal conduit formation. This is a known complication of such urinary diversion surgery, affecting up to 42% of patients [15]. The mechanism is thought to be due to the reabsorption of chloride ions from the urine in exchange for bicarbonate, when the urine comes into contact with the bowel mucosa. The significant metabolic acidosis was not picked up early enough and caused several physiological changes which accounted for many of his presenting symptoms; the feeling of breathlessness due to the tachypnoea as a compensatory response to the metabolic acidosis; worsening nausea and anorexia due to the progressive acidosis, leading to a significant reduction in oral nutrition manifesting with weight loss, anaemia and weakness. We postulate that the significant reduction in oral intake is what led to the maladaptation of the small bowel mucosa, which started reverting to normal appearance as the metabolic changes were corrected and the oral intake increased.

## Conclusions

This case provides a clinical example, with biopsy evidence, of the maladaptation of the intestinal mucosa to significantly reduced oral intake, as explained above. Furthermore, the series of repeat biopsies over a two-year period showed parallel improvement in the mucosal changes with ongoing nutritional recovery, once the metabolic acidosis which was causing the patient’s loss of appetite had been corrected.

When investigating a patient who presents with chronic loose stools and weight loss, it is obviously important to rule out coeliac disease as a cause, especially in the presence of villous atrophy. Initial anti-tissue transglutaminase IgA antibodies testing is usually enough to establish this diagnosis in the vast majority of cases. If the serology is negative, it is important to review the pathology in light of the clinical picture and consider the possibility of non-coeliac enteropathies (NCE). Investigation of serum immunoglobulins to exclude IgA deficiency, checking for IgG anti-tTg antibodies, and HLA- DQ2/DQ8 testing will provide further guidance to exclude coeliac disease. In the absence of positive findings, it is reasonable to assume a diagnosis of NCE, especially if the patient has not seen an improvement on a gluten-free diet. We have demonstrated a series of investigations, exploring the causes of villous atrophy in this gentleman, trying to correlate the clinical features. Due to the paucity of clinical literature documenting such changes in adult populations, we did not initially consider the fact that the villous atrophy in this patient may actually have been the result of maladaptation of the small bowel mucosa to malnutrition, rather the cause of the malnutrition itself.
